# Experimental Study on the Detection of Hazardous Chemicals Using Alternative Sensors in the Water Environment

**DOI:** 10.3390/toxics10050200

**Published:** 2022-04-19

**Authors:** Su-Han Nam, Tae-Geom Ku, Ye-Lim Park, Jae-Hyun Kwon, Do-Sung Huh, Young-Do Kim

**Affiliations:** 1 Department of Civil & Environmental Engineering, Myongji University, Seoul 17058, Korea; nsh3750@nate.com; 2 Department of Environmental Engineering, Gyeongnam Institute, Changwon 51430, Korea; yhku1986@hotmail.com; 3 Department of Civil and Environmental Engineering, Inje University, Gimhae 50834, Korea; pyealim@oasis.inje.ac.kr; 4 Department of Civil and Environmental Engineering, Nakdong River Environmental Research Center, Inje University, Gimhae 50834, Korea; envkwon@inje.ac.kr; 5 Department of Chemistry and Nano Science and Engineering, Center of Nano Manufacturing, Inje University, Gimhae 50834, Korea; chemhds@inje.ac.kr

**Keywords:** chemical accident, chemical spill, accident preparedness substance, alternative indicator, alternative sensor

## Abstract

Chemical accidents in rivers may be triggered by natural or anthropogenic causes and refer to the flow of large quantities of hazardous chemicals into rivers. In South Korea, domestic water is sourced from large rivers, such as the Nakdong River. However, owing to rapid industrialization, industrial facilities have become heavily concentrated in the middle and upper reaches of the Nakdong River. Therefore, severe problems could arise if harmful chemicals are leaked from industrial facilities into the river, and this contaminated river water is supplied to cities. Quantitative evaluation based on instrumental analysis during chemical accidents and prediction research based on modeling is actively being conducted however, research on the initial response is insufficient. Therefore, in this study, the variations in pH and EC were analyzed according to their chemical concentrations for seven chemicals. These seven chemicals are designated accident-preparedness substances that frequently cause chemical spills in South Korea. Additionally, we evaluated the possibility of identifying unknown substances by comparing the variations in pH and EC and statistics while diluting unknown substances. Thus, the potential of pH and EC as alternative indicators for detecting and identifying chemicals was evaluated in this study. NaF, NH_4_HF_2_, NaCN, and NH_4_OH were classified by comparing their spatial distributions in a pH-EC relation curve. However, H_2_SO_4_, HCl, and SOCl_2_ showed similar spatial distributions in the pH-EC curves and were difficult to identify. The results of this study provide information for chemical detection and identification using alternative sensors that permit easy and rapid field measurements in the event of a chemical spill and could be used as preliminary data for rapidly responding to accidents.

## 1. Introduction

Chemical accidents in rivers can occur either naturally or anthropogenically and refer to the discharge of large quantities of chemicals into rivers from factories or spills during transport or handling. Chemical accidents adversely affect ecosystems and humans through physical and chemical reactions; particularly, the unnatural quantities of chemicals entering the water system can alter the water environment [1]. Several chemical accidents have occurred worldwide, such as the leakages of sulfuric acid in Old Delhi, India in 1985; cyanide in Baia Mare in 2000; nitric acid in a port in Krefeld-Uerdingen, Germany in 2001, and marine leakages of phosphoric acid in the Marshall Islands in 2006 and sodium cyanide in the Port of Tianjin, China in 2015 [2,3,4,5,6].

The water intake rate for rivers in Korea is approximately 40%, which is high among the Organization for Economic Co-operation and Development (OECD) countries. Most of Gyeongsangbuk-do province relies on the Nakdong River for raw water [7], and the city of Daegu extracts 1.11 million tons of water from the Nakdong River, which represents 74% of the daily domestic water usage [8]. However, owing to rapid industrialization, industrial facilities have become heavily concentrated in the middle and upper reaches of the Nakdong River, the primary source of drinking water [9,10]. This suggests that severe problems could arise if harmful chemicals leak in large quantities and contaminate the raw water source due to chemical accidents, especially if subsequent measurements or predictions cannot be conducted rapidly [11]. A major chemical accident in the Nakdong River occurred on 13 March 1991; 30 tons of phenol stock from Doosan Electronics, Gyeongsangbuk-do, spilled into the mainstream of the Nakdong River over approximately 8 h [12]. Several other hazardous chemicals have also spilled into the Nakdong River, including dioxane in 2004, perchlorate in 2006, and a second phenol spill in 2008 [13]. In total, 656 water pollution accidents have occurred in South Korea between 2014 and 2018, including oil spills (313 accidents, 45.0%), fish kills (206 accidents, 29.6%), and chemical spills (61 accidents, 8.8%) [14]. Many domestic and overseas studies have been conducted for oil pollutant accidents because oil spills account for the largest percentage of water pollution accidents in rivers. Studies on oil spills typically predict the arrival time and diffusion range [15], focusing on the simulation of oil transport and diffusion using Lagrangian particle tracking (LPT) [16,17,18]. Furthermore, the Korea Environment Corporation has been striving to immediately respond to water pollution accidents by preparing disaster prevention guidelines [19].

The leakage of hazardous chemicals other than oil pollutants is analyzed through prediction models and the collection of samples for laboratory analysis. Prediction research involving modeling utilizes mathematical models that entail a predictive function for leakage [20]. During the leakage of benzene and nitrobenzene in the Songhua River, China, in 2005, models were used to predict the arrival time of pollutants and their maximum concentrations [21]. A mock scenario was also created with AQUATOX-EFDC involving the leakage of 30–30,000 kg of toluene into the Jeonju River in Korea [22]. Laboratory analysis using analytical equipment, such as gas chromatography/mass spectrometry equipment, needs to be conducted when sample collection is involved. Analysis methods for hazardous chemicals have been presented by the U.S. Environmental Protection Agency (USEPA), Occupational Safety and Health Administration (OSHA), and the National Institute of Occupational Safety & Health (NIOSH) [23,24,25,26]. Qualitative and quantitative chemical evaluation studies using these methods can produce preliminary data for investigating the environmental impacts caused by chemical accidents [27].

However, the above analytical procedure has limitations in terms of the speed of detecting and identifying chemicals in the event of an accident, making its application difficult for formulating an initial response. For a rapid response in the event of a chemical accident, the pollutant should be easy to measure. In addition, pH and electrical conductivity (EC) can be utilized as alternative indicators for the detection and identification of chemical substances if a high concentration of a chemical substance flows into the river and changes the pH and EC of the water in a unique pattern. Therefore, in this study, we selected accident preparedness substances that are frequently released because of chemical spill accidents in South Korea and analyzed the variations in pH and EC according to their concentrations. In addition, these results were used to identify other diluted unknown substances by comparing the variations in pH and EC. Finally, the possibility of using pH and EC as alternative indicators for chemical detection and identification was evaluated.

## 2. Experimental Set-Up and Procedure

### 2.1. Chemical Reagents and Measurement Sensors

The Chemical Control Act of South Korea has described and is regulating 97 accident preparedness substances. In South Korea, most industries handling chemicals are located near large rivers. In this study, we selected seven chemicals among the accident preparedness substances that are frequently released because of chemical accidents and are handled in most factories (Table 1).

The ability of alternative indicators to detect the organic matter among the accident preparedness substances presents certain limitations owing to its extremely low-dissociation rate in water.

Furthermore, when substances, such as oil or phenol enter the river, their detection is easy as they are visible to the naked eye. South Korea has already developed suitable response plans for oil and phenol. Therefore, this study only considered inorganic chemicals that are difficult to detect with the naked eye.

Sensors for measuring the changes in pH and EC were selected according to the concentrations of the analyzed chemicals. The pH was measured using a Professional pH meter PP-50 (Sartorius AG, Göttingen, Germany). The measurement range is −2 to 20, and the accuracy is ±0.002 [28]. EC was measured using YSI pro 2030 (YSI, Yellow Springs, OH, USA). The measuring ranges are 0–500 µS/cm, 0–5 mS/cm, and 0–50 mS/cm, and the resolution is changed to 0.0001 to 0.1 mS/cm, 0.1 to 0 µS/cm depending on the measuring range [29]. Table 2 shows the performances of the devices used for measuring pH and EC.

### 2.2. Selectd Solvents

Conducting field experiments to measure the concentration changes over time by introducing hazardous chemicals into natural rivers is not realistic. Therefore, solvents were selected to dilute the chemicals assuming that their concentration after entering the rivers was diluted over time at specific points. Furthermore, since the baseline pH and EC differ in each river, changes in these values may vary depending on the river into which the chemicals flow. Therefore, we selected solvents by considering the characteristics of river water and the conditions that may produce different baseline concentrations in the same river (ordinary season and flood season) (Table 3).

The Joman River (JM), located in Juchon-myeon, Gimhae-si, Gyeongsangnam-do, has agricultural and industrial areas that are located around the water-sampling point. The Sineo Stream (SS) is a waterfront river in Gimhae-si, Gyeongsangnam-do that flows through a residential area. The West Nakdong River (WNR) is a lake-type river, wherein floodgates cause the water body to stagnate, and the flow rate is controlled by the Noksan Floodgate in the estuary and the Daejeo Floodgate in the upstream section. The Gam Stream (GS), which is located in the midwestern part of Gumi-si, Gyeongsangbuk-do, accommodates several factories that handle chemicals in the Gam Stream Basin. Furthermore, large-scale chemical accidents have previously occurred around the Gam Stream.

### 2.3. Experimental Method

The concentration range for this study was selected considering high to low concentrations that are representative of chemical accidents, assuming a case of chemical flow into the river (Table 4).

A 0.5 M stock solution of the selected chemicals were prepared to improve the accuracy of low-concentration solutions. The samples were prepared by transferring 200 mL of river water into 30 measuring cylinders for each chemical and adding the stock solutions prepared at the concentrations stated in Table 4.

To conduct experiments at different baseline concentrations for each chemical, six solvents were prepared using JM, SS, and WNR water samples collected during ordinary and flood water seasons (Table 3).

The pH and EC were measured in samples with concentrations of 0–3000 mg/L of each chemical in different solvents. The pH and EC measurements were combined for each chemical depending on the concentration of the six solvents.

Subsequently, experiments were performed using the combined results to verify whether the substances can be identified when the selected chemicals flow into another river. In this experiment, high-concentration samples were produced assuming that each chemical is an unknown substance (Table 5). The pH and EC were measured after diluting the unknown samples to a low concentration using GC water as the solvent. In addition, the variations in the measured pH and EC were compared with the combined results to identify the chemicals in the unknown samples.

## 3. Results and Discussion

**Figure 1:**
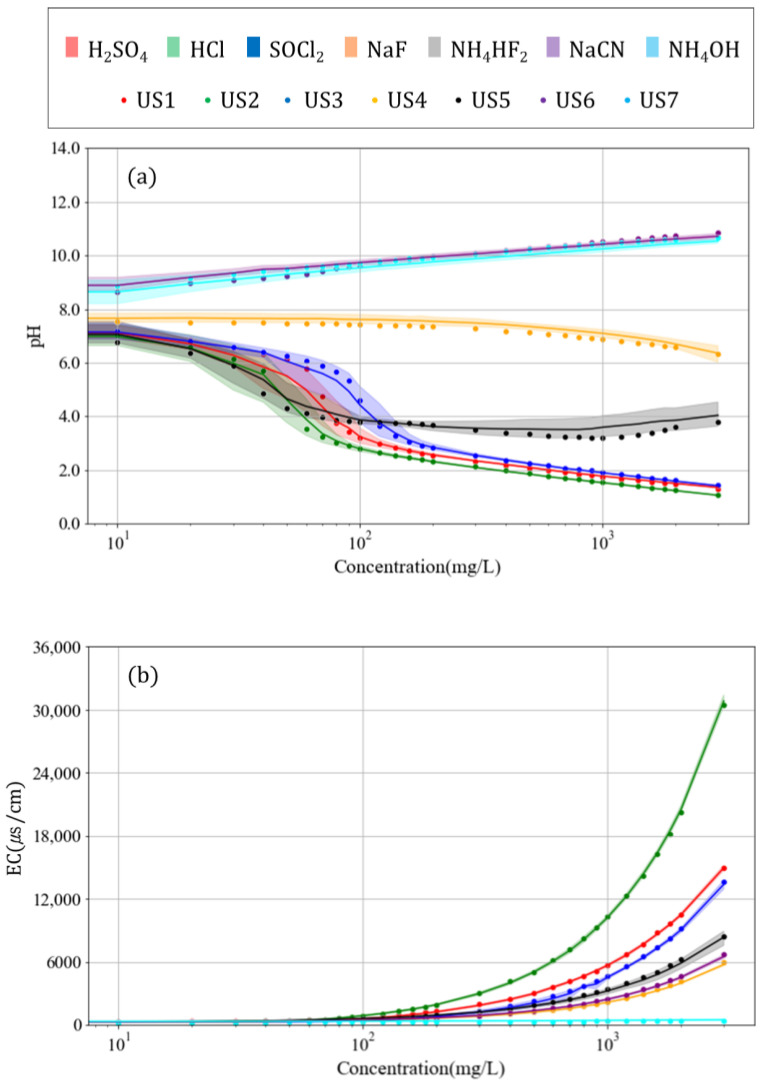
Variations in (**a**) pH and (**b**) EC with concentration of chemicals in surface water sample (log scale).

The pH and EC were measured according to the concentrations of chemicals in samples produced using six solvents. Each of the seven chemicals under the conditions described in Table 4 are shown in Table 1. The pH and EC results of the six solvents were combined and used to prepare a line plot. In addition, the pH and EC results measured after diluting the unknown substances at different concentrations were combined and plotted, as shown in Figure 1. The points representing the seven unknown chemicals appear to be included within the line plot confidence interval of each chemical, suggesting the possibility of identifying unknown chemicals.

The substances that caused the sample pH to decrease to <2, namely H_2_SO_4_, HCl, and SOCl_2_ (Figure 1a), were difficult to distinguish at concentrations higher than 100 mg/L. However, at low concentrations (<100 mg/L), the slope of decreasing pH presented a different trajectory for each substance. Furthermore, NaCN and NH_4_OH were difficult to distinguish based on pH alone because their pH values presented similar trends. However, other substances showed distinct patterns in pH.

Distinguishing substances at concentrations below approximately 500 mg/L was difficult because of the low increase in the EC with concentration (Figure 1b). However, when the concentration was >500 mg/L, the slope of increasing EC differed for each substance. The EC varied for NaCN and NH_4_OH, even though they displayed similar variations in pH. This suggests that to identify different chemicals, the simultaneous comparison of pH and EC is necessary.

**Figure 2:**
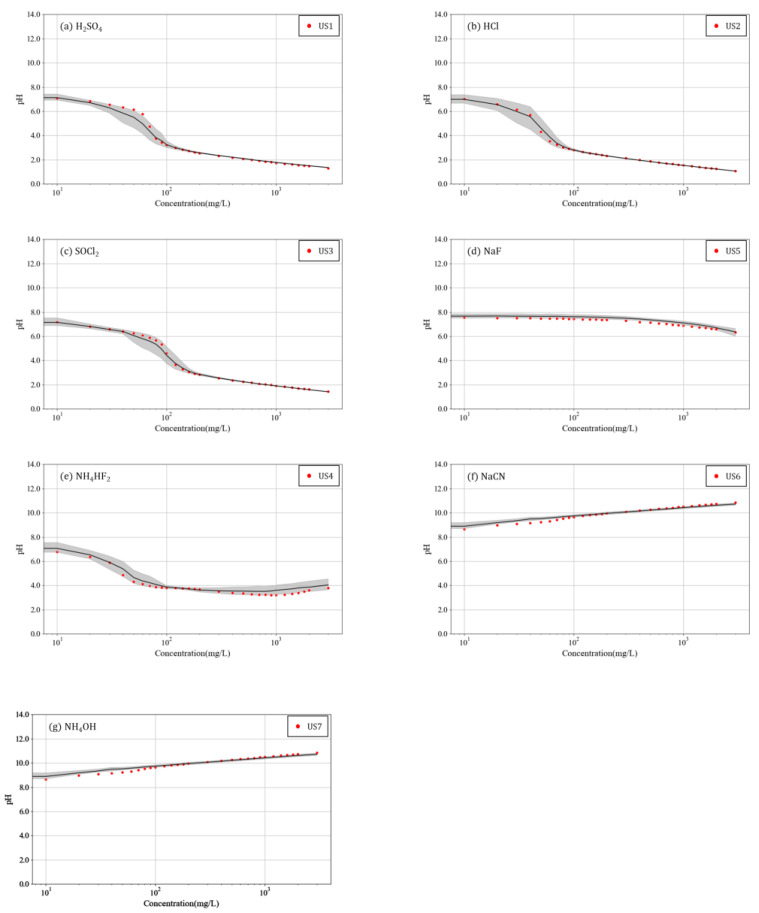
Variation in pH with concentration for several chemicals in surface water sample (log scale).

**Figure 3:**
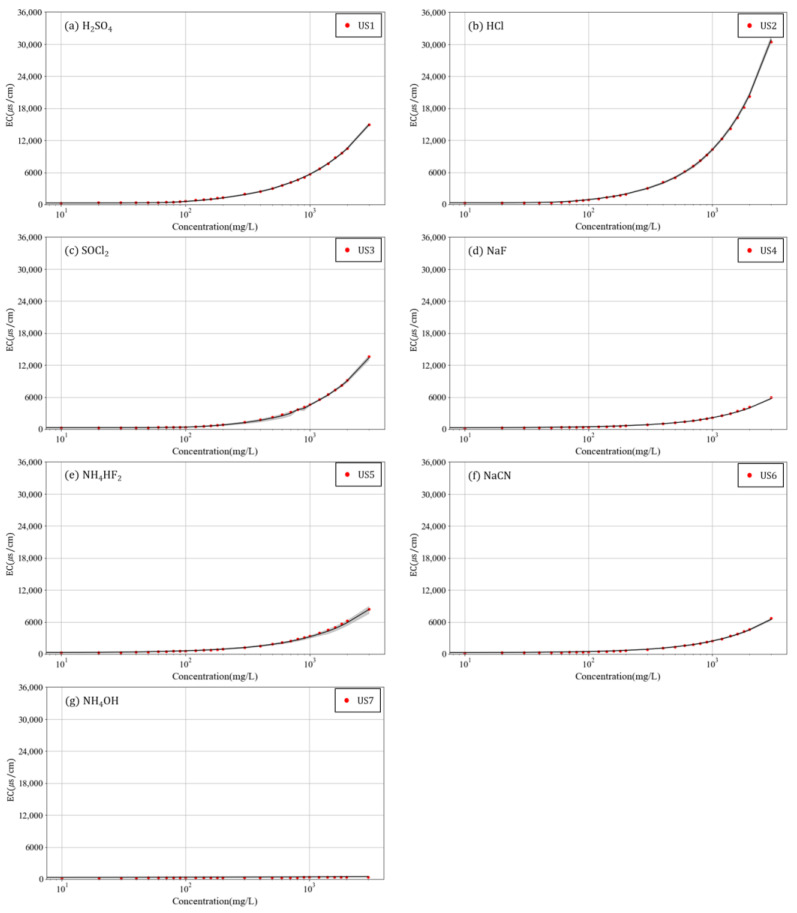
Variation in EC with concentration of several chemicals in a surface water sample (log scale).

Figure 2 and Figure 3 present an analysis of the results in Figure 1 for each chemical.

The substances in Figure 2a–c are acidic at high concentrations, with pH 2 or lower. The distribution of maximum and minimum values appears extremely narrow at concentrations where the pH is ≤3. This can indicate that at high concentrations, the pH variations in the three chemicals were negligibly affected by the baseline concentration of the river. In contrast, at concentrations <100 mg/L, where the pH decreases sharply, the distribution of the maximum and minimum values appear relatively wide. This is similar to the phenomena appearing before and after the equivalence point of the pH titration curve, which is caused by an experimental error that occurs when the pH decreases sharply.

In Figure 2d, pH remained somewhat unchanged with the concentration. In Figure 2e, the distribution of the maximum and minimum values of pH had a small range of variation. Figure 2f,g present similar increasing tendencies for pH, and the distribution of the maximum and minimum values of pH appeared insignificant.

When the pH values of the six solvents were compared with that of unknown substances at varying concentrations, they revealed similar patterns, as shown in Figure 2.

Figure 3 shows the variation in EC according to the chemical concentration. Every substance except that in Figure 3g displayed a constant increase in the EC value with an increase in concentration. This is because of the dissociation of the chemicals. As substances have a high dissociation rate when they are highly acidic, as shown in Figure 3b, the increasing slope of the EC was higher for acidic substances than that of other substances. In Figure 3g, the substance was a weak base, and ionization in water was negligible, resulting in almost no change in EC.

Furthermore, the variation in the EC values of unknown substances revealed similar patterns (Figure 3). Thus, the variations in pH and EC according to the concentration of each chemical implied that the effect of the baseline concentration of rivers is insignificant and that each chemical exhibits a unique relationship with pH and EC according to their concentration.

The unknown substances were identified by comparing the variations in pH and EC according to their concentrations with the average variations in pH and EC according to the concentrations of each chemical in the six solvents. The mean absolute percentage error (MAPE) was used for statistical comparisons and was calculated according to Equation (1):

(1) $$\mathrm{MAPE}=\;\frac{1}{n}\overset{n}{\underset{t=1}{\sum }}\left|\frac{A_{t}-F_{t}}{A_{t}}\right|$$

where $A_{t}$ is the actual value and $F_{t}$ is the forecast value.

The experimental results for the seven chemicals at six different concentrations were designated as the actual values. The results of experiments on unknown chemicals were designated as the forecast values. Table 5 shows the statistical analysis results of the two experiments. The unknown substances were identifiable based on the assumption that they displayed the lowest MAPE values for pH and EC when compared with the correct chemicals; all chemicals except NaCN and NH_4_OH showed distinctly lower MAPEs for both pH and EC than those of other substances. However, NaCN and NH_4_OH were still considered identifiable because their MAPE values for EC distinctly differed from each other, except for the low MAPEs at pH < 2. The unknown substances identified based on the statistical results matched the seven unknown substances listed in Table 5.

The abovementioned results confirm that each chemical flowing into the river exhibited a unique pH and EC variation pattern according to its concentration. Therefore, the pH and EC have the potential to be considered alternative indicators for identifying unknown substances in the event of chemical accidents.

**Figure 4:**
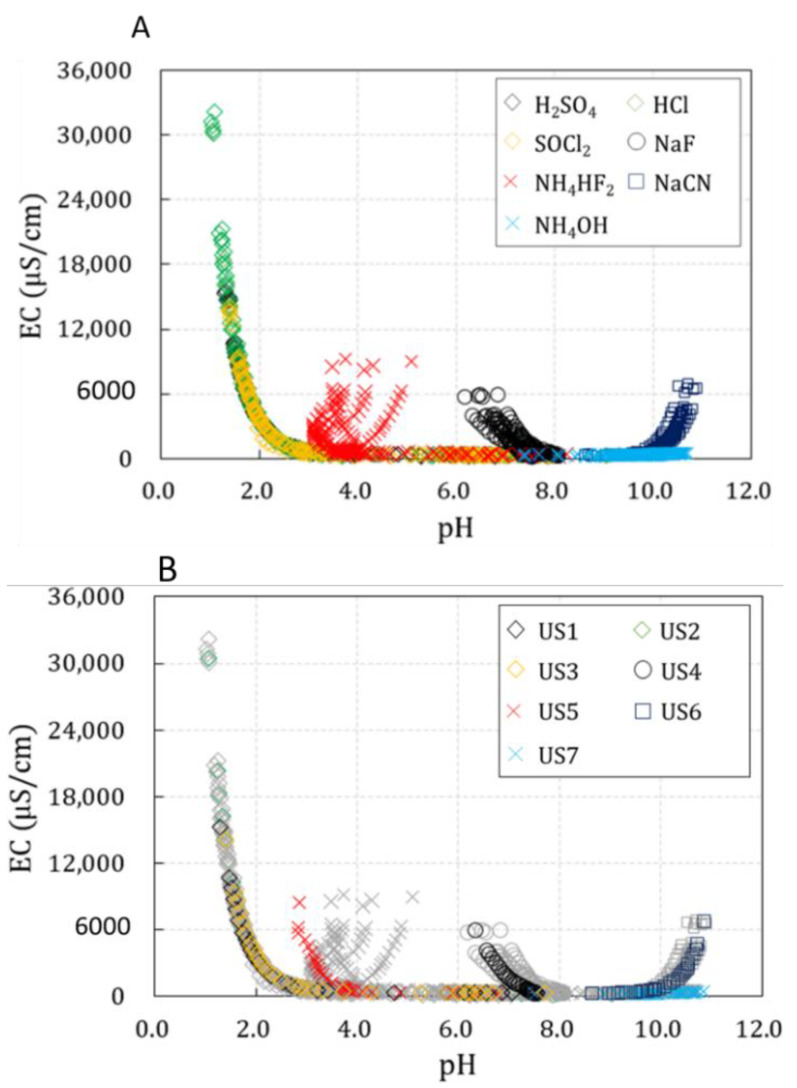
pH-EC relation curve. (**A**) Known chemicals; (**B**) Known and unknown chemicals.

However, when chemicals enter a river and alternative indicators are measured at a specific point, the total concentrations of these chemicals cannot be determined. Therefore, the variations in the alternative indicators measured in this study according to the chemical concentrations were combined and represented as pH-EC relation curves (Figure 4). We attempted to evaluate whether these alternative indicators can be used in natural rivers to identify chemical accidents. When a large-scale chemical accident occurs in a river, a high concentration of unknown chemicals enters the river water and is diluted to a low concentration. When an experiment was conducted assuming this, each unknown chemical showed a similar tendency to the corresponding known chemical, allowing their identification.

Figure 4a shows a pH-EC relation curve prepared using the pH and EC values of six solvents for each chemical. Since each chemical showed different patterns for the alternative indicators as shown in Table 5, the pH-EC relation curves presented different characteristics.

However, the substances with pH < 2 at high concentrations, namely H_2_SO_4_, HCl, and SOCl_2_, had very similar distributions in the pH-EC relation curve. Conversely, the remaining substances (NaF, NH_4_HF_2_, NaCN, and NH_4_OH) had distinct distributions.

Figure 4b shows the measurement results of the seven unknown chemicals based on the results shown in Figure 4a, along with those of the selected chemicals. NaF, NH_4_HF_2_, NaCN, and NH_4_OH could be clearly classified by comparing the distributions of unknown substances in the pH-EC relation curve. However, as H_2_SO_4_, HCl, and SOCl_2_ showed similar spatial distributions in the pH-EC relation curve, identifying them in natural rivers using the alternative indicators was difficult.

This study is a basic research stage on chemical accidents, and research was conducted using an accident preparedness substance designated in Korea. For the detection and identification of chemicals, pH and EC were used as alternative indicators, and the possibility of use was confirmed when the two indicators were linked. In Figure 4b using the pH-EC relation curve, it can be seen that unknown chemical substances have similar trends and spatial distribution to the corresponding chemical substances. The selected alternative indicators are simple and easy to measure, so they can be used as basic data for initial response in the event of a chemical accident. However, this study was limited to individual inorganic chemical substances; therefore, the detection and identification of complex chemicals and organic chemicals need further research. In addition, other factors, such as algal blooms, river deposits, and non-point pollution sources, can alter the pH and EC of the river; consequently, research on indicators other than pH or EC that can readily detect chemicals is necessary.

## 4. Conclusions

An experimental study on the variations in pH and EC according to the concentrations of chemicals was conducted assuming the inflow of hazardous chemicals into a river. Since field experiments in rivers using hazardous chemicals are not realistic, the experimental conditions were designed assuming that the concentrations of chemicals will be diluted over time as they enter and flow in the river. Considering that each river has different base concentrations, river water samples were collected during the ordinary and flood seasons at three points and used as solvents for the chemicals, and a sufficiently high concentration range was selected to represent chemical accidents. Furthermore, the possibility of identifying unknown substances was evaluated through statistical analysis of the measurement results and unknown substances. The results demonstrated the possibility of using pH and EC as alternative indicators for chemical detection and identification. The findings of this study can be summarized as follows:

(1) The measurement results for pH and EC according to the concentration of the seven chemicals showed very similar patterns, even when solvents with different base concentrations were used. This confirmed that the variations in pH and EC according to the concentrations of chemicals had low sensitivity to the base concentration of the solvent and that each chemical has a distinct effect on these parameters.
(2) For each chemical, the average variations in pH and EC in six solvents according to the chemical concentrations were numerically compared with the variations in pH and EC according to the concentrations of unknown substances. The unknown substances were identified by assuming that the MAPEs of pH and EC would be the lowest when comparing the unknown substance with the correct chemical. Consequently, all seven unknown substances could be identified through the MAPEs, demonstrating the possibility of using pH and EC to identify chemicals.
(3) The actual concentrations of chemicals cannot be obtained by measuring pH and EC in the event of chemical accidents in rivers. Therefore, the measurement results according to the concentrations of the seven chemicals were combined and represented as pH-EC relation curves. NaF, NH_4_HF_2_, NaCN, and NH_4_OH could be clearly classified by comparing their distributions in a pH-EC relation curve. However, H_2_SO_4_, HCl, and SOCl_2_ showed similar distributions in the pH-EC relation curve. Therefore, there are limitations in classifying these three substances using pH and EC in natural rivers.

This study shows that chemicals can be detected and identified in the field using alternative sensors with easy and fast measurements in the event of chemical spills. However, there were limitations in identifying specific chemicals using these indicators, and the quantitative evaluation of chemicals was impossible. It is expected that faster chemical detection and identification may be possible by combining the results of this study with data-based research techniques, such as deep learning if the information on the pH-EC relation curves of accident preparedness substances can be produced in advance. Furthermore, the findings of this study could be used as basic data to develop the initial response plan for chemical accidents.

## Figures and Tables

**Table 1:** Selected hazardous chemicals.

Name	CAS No.	Molecular Formula	Molecular Weight (g/mol)	Number of Factories Using Substance	Chemical Family
Sulfuric acid	7664-93-9	H_2_SO_4_	98.09	2588	Inorganic oxidizing acids
Hydrochloric acid	7647-01-0	HCl	36.46	3058	Inorganic non oxidizing acids
Thionyl chloride	7719-09-7	SOCl_2_	118.97	40	Acid halides
Sodium fluoride	7681-49-4	NaF	41.99	162	Inorganic compounds
Ammonium bifluoride	1341-49-7	NH_4_HF_2_	57.04	385	Inorganic compounds
Sodium cyanide	143-33-9	NaCN	49.01	673	Inorganic cyanides
Ammonia hydroxide	1336-21-6	NH_4_OH	35.05	963	Bases

**Table 2:** pH and EC of selected rivers.

River	pH	EC (µS/cm)
Joman River (JM) (Dry Season/Wet Season)	7.5/7.4	335.6/308.2
Sineo Stream (SS) (Dry Season/Wet Season)	8.6/7.6	234.1/158.5
West Nakdong River (WNR) (Dry Season/Wet Season)	8.7/8.0	350.2/297.0
Gam Stream (GC)	9.9	212.4

**Table 3:** Range of measured concentrations.

Range (mg/L)	0–100	100–200	200–1000	1000–2000	2000–3000
Concentration Interval (mg/L)	10	20	100	200	1000

**Table 4:** Selected unknown substances.

	US1	US2	US3	US4	US5	US6	US7
Unknown Substance	H_2_SO_4_	HCl	SOCl_2_	NaF	NH_4_HF_2_	NaCN	NH_4_OH

**Table 5:** Statistical analysis (MAPE) of the estimation of unknown chemicals.

US1	H_2_SO_4_	HCl	SOCl_2_	NaF	NH_4_HF_2_	NaCN	NH_4_OH
pH	2.81%	16.43%	9.95%	57.40%	34.23%	67.23%	66.51%
EC	6.59%	31.34%	38.16%	99.22%	45.69%	82.88%	698.12%
US2	H_2_SO_4_	HCl	SOCl_2_	NaF	NH_4_HF_2_	NaCN	NH_4_OH
pH	12.77%	1.85%	21.61%	63.24%	37.89%	71.70%	71.08%
EC	56.46%	3.74%	103.43%	216.29%	125.18%	187.16%	1320.96%
US3	H_2_SO_4_	HCl	SOCl_2_	NaF	NH_4_HF_2_	NaCN	NH_4_OH
pH	14.12%	32.38%	1.94%	52.17%	33.29%	63.25%	62.44%
EC	19.23%	45.55%	5.11%	59.92%	31.26%	48.28%	550.95%
US4	H_2_SO_4_	HCl	SOCl_2_	NaF	NH_4_HF_2_	NaCN	NH_4_OH
pH	187.13%	234.66%	159.90%	2.43%	79.34%	27.94%	26.48%
EC	41.18%	58.29%	28.48%	4.92%	29.75%	9.71%	239.25%
US5	H_2_SO_4_	HCl	SOCl_2_	NaF	NH_4_HF_2_	NaCN	NH_4_OH
pH	54.46%	75.09%	48.51%	46.80%	5.41%	60.32%	59.50%
EC	26.34%	43.99%	26.53%	40.64%	5.67%	30.24%	401.27%
US6	H_2_SO_4_	HCl	SOCl_2_	NaF	NH_4_HF_2_	NaCN	NH_4_OH
pH	316.95%	387.18%	278.91%	36.15%	151.80%	1.20%	1.70%
EC	39.43%	57.58%	25.38%	11.25%	26.14%	6.49%	274.75%
US7	H_2_SO_4_	HCl	SOCl_2_	NaF	NH_4_HF_2_	NaCN	NH_4_OH
pH	315.87%	385.83%	277.79%	36.45%	152.09%	0.19%	1.90%
EC	69.15%	75.80%	62.12%	59.70%	69.06%	61.32%	2.34%

Note: The Unknown Substance (US) is the same chemical as the underlined.
